# Regulation of Protein Synthesis at the Translational Level: Novel Findings in Cardiovascular Biology

**DOI:** 10.3390/biom15050692

**Published:** 2025-05-09

**Authors:** Sergey Tsoy, Jiandong Liu

**Affiliations:** 1 Medical Scientist Training Program, University of North Carolina at Chapel Hill, Chapel Hill, NC 27599, USA; sergey_tsoy@med.unc.edu; 2 McAllister Heart Institute, University of North Carolina at Chapel Hill, Chapel Hill, NC 27599, USA; 3 Department of Pathology and Laboratory Medicine, University of North Carolina at Chapel Hill, Chapel Hill, NC 27599, USA

**Keywords:** translation, hypertrophy, cardiovascular diseases, cardiomyocyte, protein synthesis, ribosomes

## Abstract

Translational regulation plays a pivotal role in cardiac gene expression, influencing protein synthesis in response to physiological and pathological stimuli. Although transcription regulates gene expression, translation ultimately determines protein levels, making it a crucial research focus. In cardiomyocytes, disruptions in this process contribute to various cardiac diseases, including hypertrophy, fibrosis, dilated cardiomyopathy, ischemic heart disease, and diabetic cardiomyopathy. Emerging evidence highlights the significance of translational regulation across multiple cardiac cell types, such as cardiomyocytes and fibroblasts, and its role in disease progression. During cardiac remodeling, transcriptomic changes are often modest, suggesting that post-transcriptional mechanisms, particularly translation, play a dominant role in cellular adaptation. This review discusses key methodologies for studying translational regulation and novel mechanisms of translational regulation related to different cardiac pathologies and highlights relevant therapeutic avenues for targeting these pathways.

## 1. Introduction

Protein synthesis is a critical component of gene expression. While the analysis of mRNA levels has traditionally been the dominant method for assessing cellular gene expression, the importance of translational regulation has gained increasing recognition in recent years. Transcription provides the blueprint for protein production, but it is translation that determines the actual output [1]. Various regulatory mechanisms at the translational level fine-tune protein synthesis, which is why relying solely on transcriptional data as a proxy for protein expression can be misleading [2]. Factors such as mRNA stability, localization, and the efficiency of translational initiation can all significantly influence protein synthesis. Translational regulation is essential for maintaining cellular homeostasis, growth, and development [3]. Furthermore, since many eukaryotic mRNAs have long half-lives, modifying translational efficiency becomes necessary for rapid adjustments in protein levels in response to cellular demands [4]. Disruptions in this tightly regulated process are implicated in a variety of diseases, making translational control a critical area of study.

In the heart, translational regulation contributes to significant structural remodeling and functional adaptation in response to physiological and pathological stimuli. Cardiomyocytes, which account for 70% of the volume of the heart, are the major cell type participating in this response and are therefore a major contributor to protein level transformations seen in diseased states [5]. These metabolically active cells rely heavily on protein synthesis to maintain contractile function and structural integrity, and must swiftly respond to fluctuations in mechanical stress, oxygen availability, and metabolic changes. Translational regulation plays a vital role in cardiomyocyte homeostasis by modulating protein synthesis to meet these demands [6]. Additionally, non-myocyte cells, such as fibroblasts, also undergo translational shifts that contribute to disease progression [7]. Increasing evidence highlights the critical role of translational regulation across multiple cardiac cell types in modulating protein synthesis, underscoring its importance in both health and disease.

Cardiac tissue is known for its low level of gene transcription compared to other organs. During pathological remodeling, despite significant structural and functional alterations in the heart, changes at the transcriptomic level are often modest [8]. This has led many researchers to suggest that cardiomyocytes rely heavily on post-transcriptional regulation, particularly at the translational level [6,7,9]. Translational regulation in the heart is involved in several key steps: (1) pre-translational setup, which includes the synthesis and spatial localization of translational machinery, (2) translational initiation, (3) translational elongation, and (4) translational termination. In cardiac diseases, these post-transcriptional mechanisms are often key drivers of the cellular adaptations needed to cope with mechanical stress or injury. In this review, we will outline the primary methodologies used to study translational regulation in the heart as well as recent findings. Particular emphasis will be placed on cardiac hypertrophy, as translational regulation has been predominantly studied in this area. Novel mechanisms relevant to fibrosis, dilated cardiomyopathy, ischemic heart disease, and diabetic cardiomyopathy will also be explored. Additionally, we will address translational regulation in the context of reprogramming of cardiac fibroblasts into induced cardiomyocytes and discuss potential therapeutic strategies with clinical relevance. By doing so, we hope to shed light on the complex landscape of translational regulation in the heart, emphasizing its potential as a therapeutic frontier.

## 2. Main Methods of Translational Study

**Figure 1:**
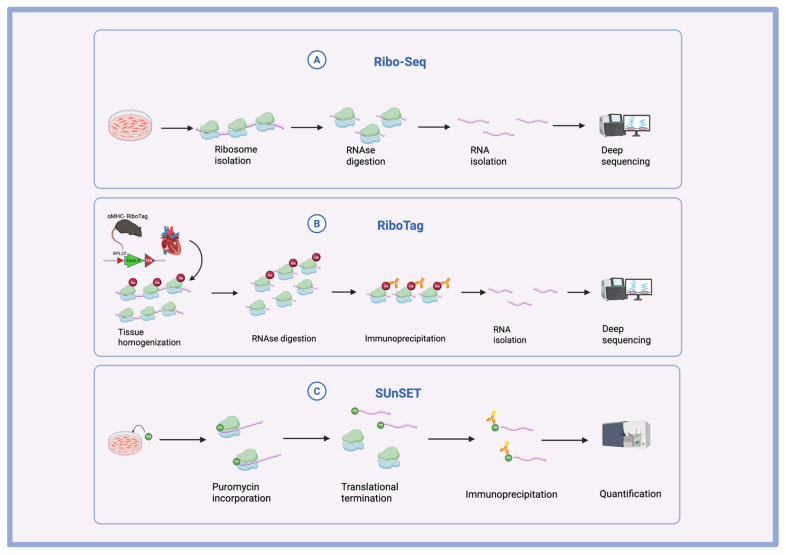
Main methods of translational study in the heart. (**A**) Summary of Ribo-Seq. Ribosome-protected RNA fragments are digested and sequenced to detect ribosomal footprints. (**B**) Summary of RiboTag. The cardiomyocyte-specific promoter drives the expression of Cre, inducing the expression of a cell-type-specific HA-tagged RPL22 ribosomal protein. Anti-HA antibodies are used to separate the labeled ribosomes, and RNA is isolated for deep sequencing. (**C**) Summary of SUnSET Assay. Cells are incubated with puromycin. Translating mRNA is tagged with puromycin, and anti-puromycin antibodies are used for the quantification of signals.

Various methodologies have been established to study translational regulation, each with different levels of specificity. The SUnSET assay quantifies global protein synthesis, making it useful for studying translational regulation, especially in gene knockdown or knockout experiments. When it comes to delineating whole translatome shifts under varying physiological and pathological changes, ribosome profiling is a key tool in translational studies. When combined with mRNA sequencing, it allows for the normalization of data to determine translational efficiency, offering insights into dynamic translational regulation. Additionally, the application of these methods to in vivo models, such as the Ribo-Tag mouse, facilitates the selective tagging of ribosomes in specific cell types, enabling mid-translational isolation with minimal disturbance to cellular integrity (Figure 1). Together, these techniques provide a powerful framework for investigating the complexities of translational control in cardiovascular biology.

The SUnSET assay, involving puromycin pulsing, is a relatively simple but effective method for measuring global translation activity. Puromycin, an aminoacyl-tRNA analogue, is incorporated into nascent peptides during active translation and inhibits elongation. Immunodetection of puromycin can provide a snapshot of global protein synthesis in cells [10]. This technique enables the quantification of translation at the cellular level without requiring the detailed mapping of individual ribosome positions.

Ribosome profiling quantifies active translation by sequencing mRNA fragments protected within ribosomes, offering insights into translation efficiency. In this method, RNases are used to degrade unprotected mRNA fragments, while ribosome-protected fragments (RPFs) are retained. These RPFs can then be aligned to a reference genome, pinpointing the exact locations of ribosomes on the transcript. This provides a detailed “footprint” of translation for specific mRNAs [11]. The locations where translation is slow are overrepresented in the sequencing data, as ribosomes spend more time at those positions [12]. The nucleotide resolution provided by Ribo-Seq is a powerful advantage over polysome profiling.

When coupled with RNA-seq, Ribo-Seq allows for the calculation of translational efficiency (TE), a metric that compares ribosome occupancy with mRNA abundance. This approach provides a dynamic view of translation at a genome-wide scale. More advanced applications, such as the ΔTE value, have been developed to measure fluctuations in translational efficiency over time or in response to changing cellular conditions, offering a more detailed analysis of how translation adapts to various physiological or pathological stimuli [13].

A major advantage of ribosome profiling over other methods is its ability to detect novel open reading frames (ORFs), such as upstream ORFs (uORFs) [14]. UORFs are short sequences of nucleotides located in the 5′ untranslated region (UTR) of an mRNA that can initiate translation before the main coding sequence (the primary ORF). uORFs can regulate the translation of the downstream main ORF by either inhibiting or modulating the ribosome’s ability to re-initiate translation after translating the uORF [15]. This allows for fine-tuning of gene expression, often in response to environmental or cellular conditions, and plays a significant role in the post-transcriptional regulation of protein synthesis.

Understanding the role of uORFs in translational regulation can be achieved by inhibiting their translation. One approach is the use of antisense oligonucleotides, which can specifically bind to the start codon of uORFs and prevent ribosome loading, thus preventing translation. This method is particularly valuable for studying the regulatory function of uORFs in modulating the translation of downstream coding regions, providing insights into how uORFs contribute to the modulation of gene expression under different physiological or stress conditions [16,17].

To study translational regulation in vivo, researchers can employ models such as the Ribo-tag mouse. This model involves mice expressing a ribosomal protein tagged with an epitope, such as hemagglutinin (HA), which can be selectively activated in specific cell types using Cre recombinase. Once tagged, ribosomes can be isolated from the target cells through immunoprecipitation, and the associated translating mRNAs can be sequenced to gain insight into the translational landscape of these specific populations [18]. Another related technique, Translating Ribosome Affinity Purification (TRAP), similarly targets ribosomes from specific cell types within a tissue, but instead fuses GFP to a ribosomal subunit component, allowing for the isolation of ribosomes from specific cells via GFP immunoprecipitation [19].

## 3. Translational Regulation of Cardiac Hypertrophy

The mammalian heart can experience a dramatic size and shape transformation in response to an increased workload [20]. The lack of proliferative capacity among cardiomyocytes, however, necessitates an increase in the size of individual cells to produce a structural expansion. This process is known as cardiac hypertrophy. The two main types of hypertrophy are physiological and pathological. While both types initially develop as an adaptive response to cardiac stress, they differ greatly in long-term progression. Physiological hypertrophy, which occurs with intensive physical training, enhances cardiac function and allows the heart to more efficiently pump blood. In contrast, pathological hypertrophy occurs with conditions like hypertension and fails to sustain heart function, potentially progressing to heart failure [21].

One of the defining features of both forms of cardiac hypertrophy is the rapid accumulation of newly synthesized proteins [22]. This rapid rise in protein production is vital for cardiomyocytes to adapt to mechanical stress, given that the baseline rate of protein synthesis in the adult heart is relatively low compared to other tissues [8,23,24]. Despite the robust transformation in cardiomyocyte shape and size, transcriptomic alterations are minimal. This aligns with the growing understanding that multiple layers of regulation exist between transcription and protein synthesis, leading to a disconnect between RNA sequencing data and the proteome [25]. In contrast, translational control has emerged as a more reliable predictor of protein levels. Studies have shown that both global and selective translational levels increase significantly during hypertrophy [26,27]. These findings have placed translational regulators at the forefront of new discoveries in cardiac hypertrophy mechanisms.

Until recently, the regulation of protein synthesis at the translational level in cardiac hypertrophy remained largely unexplored. However, recent studies have shown that hypertrophy involves translationally regulatory mechanisms at multiple stages of protein synthesis. Once mRNA is transcribed, the first challenge is forming a translational complex. Cardiomyocytes can regulate translation at this stage by leveraging their microtubule network to spatially organize ribosomes and transcripts, either promoting or inhibiting translation [28]. Additionally, the extent of protein folding stress within the cell can influence how regulators such as IRE1α modulate the assembly of the translation initiation complex [29]. Even after contact has been made between mRNA and a ribosome, the presence of an upstream Open Reading Frame (uORF) can downregulate translation of the main ORF [17]. Furthermore, the supply availability of proteins that aid in translational initiation, such as PABPC1, also serves as a limiting factor [30]. Additionally, regulators like mTORC1 can play dual roles of promoting interactions between translational components during initiation, while also providing a second layer of regulation at elongation through downstream effectors [7]. These greatly differing levels of translational regulation have been shown to play important roles in cardiac hypertrophy. By unraveling the complexity of this dynamic process, we can gain a deeper understanding of the pathological progression of cardiac disease, paving the way for targeted therapies to treat or alleviate symptoms in patients.

### 3.1. Translational Complex Components in Hypertrophy

**Figure 2:**
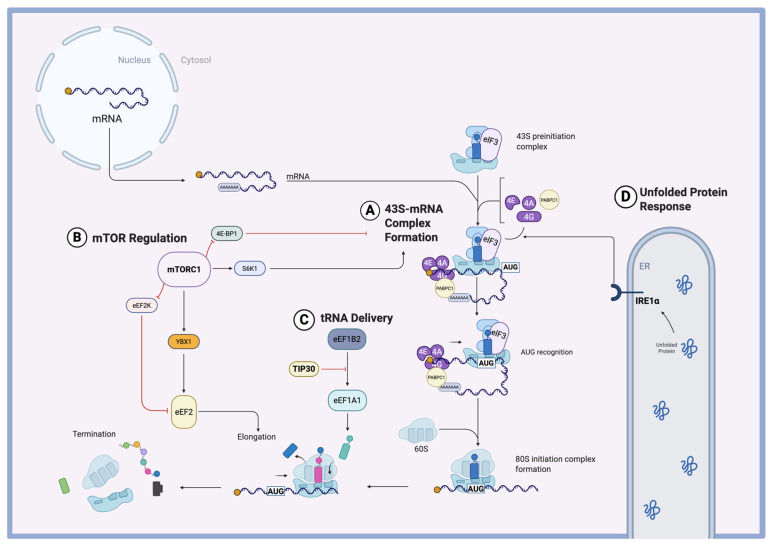
Global regulation of protein synthesis. (**A**) 43S-mRNA complex formation. Translational initiation is regulated by interactions between PABPC1 and initiation factors. (**B**) mTOR regulation. mTORC1 regulates both initiation complex formation and translational elongation. (**C**) tRNA delivery. TIP30 regulates elongation by delivering tRNA aminoacyl tRNAs to the translating ribosome. (**D**) Unfolded protein response. IRE1α regulates interactions between eIF4G and eIF3.

RNA translation is a metabolically intensive process that requires the combination of an mRNA template, aminoacyl-tRNAs, ribosomes, and translational factors. These pre-initiation factors include eukaryotic initiation factor 4F (eIF4F) along with poly(A)-binding protein (PABPC1). The eIF4F complex consists of eIF4E, which recognizes the 5′ cap, eIF4A, which functions as an RNA helicase, and eIF4G, a scaffold protein that interacts with poly(A)-binding protein C1 (PABPC1). This interaction between eIF4G and PABPC1 (Figure 2A) is necessary for the formation of a stable, looped mRNP complex, which stimulates cap-dependent translation while protecting the mRNA from exonucleases [31].

PABPC1 is an RNA-binding protein that has been studied in the context of various cancers and has now also been implicated as having a central role in mRNA translation and stability [32]. Recent studies have shown that the protein plays a pivotal role in pathological cardiomyocyte hypertrophy induced by transverse aortic constriction (TAC), a common experimental model of pressure overload. TAC surgery involves surgically narrowing the transverse aorta in animal models, which creates increased pressure in the left ventricle, mimicking the mechanical stress associated with conditions like hypertension and aortic stenosis in humans [33]. Within this model, disrupting the interaction between PABPC1 and eIF4G significantly reduces protein synthesis and attenuates pathological hypertrophy [34]. Additionally, while PABPC1 protein levels rise dramatically, the mRNA levels exhibit only modest increases from baseline to pathological states. This discrepancy can be explained by translational efficiency, which is closely linked to changes in the poly(A) tail length of *Pabpc1* mRNA. In adult cardiomyocytes, *Pabpc1* poly(A) tail length is reduced compared to fetal cells, but this modification is restored during both physiological and pathological hypertrophy [30]. This emphasizes how expression levels of translational complex components, such as PABPC1, are regulated in the heart’s adaptive response to hypertrophic stress. By controlling the availability of key initiation factors, cardiomyocytes can modulate global protein synthesis, thereby driving the cellular remodeling processes that underpin hypertrophy. Understanding the mechanism by which cardiomyocytes regulate poly-A tail length would be the next step toward the discovery of therapeutic targets.

### 3.2. Translational Initiation in Hypertrophy

Eukaryotic translation can be divided into four main stages: (1) initiation, (2) elongation, (3) termination, and (4) ribosome recycling. While the initiation of translation is the rate-limiting step in protein synthesis, translational regulators in the heart have been shown to exert control over both the initiation and elongation phases [6]. One of these central regulators is the mammalian target of rapamycin complex 1 (mTORC1), a kinase involved in nutrient-sensing pathways that promote cell growth and proliferation [35]. It is the allosteric target of the drug rapamycin, which is clinically used in cardiology and oncology. MTORC1 primarily regulates the initiation of translation through two key substrates: S6 kinase 1 (S6K1) and 4E-binding protein 1 (4E-BP1) (Figure 2B). Phosphorylation of S6K1 promotes the assembly of the translational initiation complex. This includes pre-initiation factors like eIF4A, eIF4E, and eIF4G, along with poly(A)-binding protein (PABPC1), which together form the mRNA–ribonucleoprotein (mRNP) complex required for the recruitment of the 40S ribosomal subunit. Meanwhile, mTORC1-mediated phosphorylation prevents inhibitory interaction between 4E-BP1 and eukaryotic initiation factor 4E (eIF4E) [36]. Additionally, mTORC1 selectively increases the translation of mRNAs containing 5′ terminal oligopyrimidine (TOP) motifs, which often encode components of the translational machinery [37].

Another mechanism involved in hypertrophy is the Unfolded Protein Response (UPR), a cellular stress response that helps maintain endoplasmic reticulum homeostasis by managing protein folding stress [38]. In hypertrophying cardiomyocytes, the accumulation of nascent proteins around the ER has been shown to activate the UPR, specifically involving the ER-anchored sensor protein IRE1α [29]. IRE1α coordinates the assembly of the translational initiation complex through interactions with eIF3 and eIF4G (Figure 2D), facilitating increased protein synthesis and cardiomyocyte growth. Notably, depletion of IRE1α reduced hypertrophy induced by both phenylephrine stimulation and transverse aortic constriction (TAC) surgery [29]. Interestingly, like mTORC1, this complex was found to selectively promote the translation of genes with a 5′ TOP motif [29]. Among the proteins upregulated via this pathway was EGFR, a key upstream regulator of the ERK pathway, which drives cell growth and proliferation [39]. These findings reveal a novel UPR-mediated mechanism through which cardiomyocytes can enhance the translation of 5′ TOP-containing mRNAs during hypertrophic remodeling, linking a stress-adaptive response to growth-related translational control.

### 3.3. Translational Elongation in Hypertrophy

The global regulation of protein synthesis in cardiomyocytes by mTORC1 has been demonstrated by multiple groups [36,40]. However, the specific downstream mechanisms by which mTORC1 drives pathological protein synthesis in cardiomyocytes remain incompletely understood [37]. Recent studies have begun to elucidate some of these mechanisms. Varma et al. identified a regulatory network in which mTORC1 modulates RNA-binding proteins, such as Y-box binding protein 1 (YBX1), in response to pressure overload [41]. YBX1 is an RNA- and DNA-binding protein involved in transcription, translation, and mRNA stability across various tissues. It supports cell growth and survival, and its upregulation has been implicated in cancer [42]. YBX1 showed strong upregulation at the protein level following transverse aortic constriction, despite relatively stable mRNA levels, highlighting its regulation at the translational level. Depletion of *Ybx1* significantly reduced pathological hypertrophy and improved cardiac function in vivo, in part by limiting the translation of proteins driving hypertrophic growth. YBX1 was found to enhance the translation of eEF2, a GTP-binding protein critical for the translocation of peptidyl-tRNA during elongation (Figure 2B). Furthermore, mTORC1 inhibits eEF2 kinase (eEF2K), an inhibitor of eEF2, thus further promoting elongation [41]. This finding highlights the role of mTORC1 in driving translational elongation, an underexplored but crucial aspect of translational regulation during hypertrophy.

In addition to mTORC1-dependent pathways, certain regulators act independently of mTORC1 to control translation. One example is TIP30, a tumor suppressor protein with functions in controlling cell growth, apoptosis, and cellular stress responses in multiple tissues [43]. TIP30 has been found to restrict cardiac hypertrophy by inhibiting elongation factor 1A1 (eEF1A1). TIP30 binds eEF1A1, preventing its interaction with its guanine nucleotide exchange factor (GEF) eEF1B2, thereby reducing the generation of active GTP-bound eEF1A1 (Figure 2C). This inhibition disrupts the delivery of aminoacyl-tRNAs to the ribosome, effectively slowing translational elongation and protein synthesis [44,45]. By acting independently of mTORC1, TIP30 provides an additional layer of regulation to fine-tune the hypertrophic response.

Although a mechanism for mTORC1 activation in hypertrophy has not been fleshed out, some speculations have been made. The increased demand for protein synthesis during hypertrophy places a burden on the protein-folding machinery within cardiomyocytes. It has been suggested that this need for enhanced protein folding may activate the endoplasmic reticulum (ER) stress pathway, involving transcription factors such as ATF6. Some studies have linked ATF6 activation to mTORC1 signaling via Ras homolog family member A (RhoA), although this connection has yet to be fully characterized in the heart [46]. This therefore remains an area of much-needed exploration. By pinpointing the pathways that trigger mTORC1 activation, we can explore potential intervention points to modulate its downstream translational activity and limit adverse cardiac remodeling.

### 3.4. The Distinction Between Pressure Overload and Adrenergic Stress Hypertrophy

The two main methods used for modeling cardiac hypertrophy are transverse aortic constriction (TAC) surgery and administration of phenylephrine. TAC surgery is often used in vivo to induce pressure overload hypertrophy. Phenylephrine (PE) can be used both in vitro and in vivo, but studies of translational regulation in the heart have mostly applied the method to cell culture models to mimic adrenergic stress [28,47]. While both models are valuable for elucidating the mechanisms of hypertrophy, their effects on the cardiomyocyte translatome may differ. The distinction is crucial for understanding how different stressors trigger unique hypertrophic responses at the translational level.

While both methods have been shown to impact translation, emerging evidence suggests they do so through distinct mechanisms. For instance, studies on pressure overload-induced hypertrophy, such as those examining Y-box binding protein 1 (YBX1) and other RNA-binding proteins (RBPs), have highlighted changes in translational efficiency, particularly under conditions of mechanical stress [41]. Conversely, one group found that phenylephrine-induced hypertrophy is characterized by an increase in the translation of ribosomal proteins rather than a global increase in translational efficiency [47].

When applying ribosome profiling (Ribo-seq) and RNA sequencing (RNA-seq) to calculate translational efficiency in PE-induced hypertrophy, Yan et al. found that most changes in translational efficiency were buffered by transcriptional alterations. A notable exception was an increase in the translational efficiency of ribosomal proteins, such as RPL35 and RPS3. This suggests that hypertrophy in response to adrenergic stress may be more dependent on an increase in ribosome biogenesis and protein abundance rather than a change in the overall efficiency of translation. In fact, when comparing control cells to those treated with phenylephrine, only a few protein-coding genes exhibited significantly altered translational efficiency, and many of these were related to muscle contraction rather than canonical hypertrophic pathways such as PI3K/AKT or MAPK3 [47].

Interestingly, some of the muscle contraction-related genes, such as tubulin components (*Tuba1a*, *Tuba1b*, *Tuba4a*), may still play a role in hypertrophic remodeling. This is particularly relevant considering the study by Scarborough et al., which explored the role of microtubules in phenylephrine-induced hypertrophy. Their work revealed that proper spatial organization of the translational machinery within the cell is essential for efficient protein synthesis during hypertrophic growth. Disrupting microtubules with colchicine did not affect global protein synthesis, as measured using puromycin assays, yet it prevented cardiac hypertrophy in phenylephrine-treated mice [28].

**Figure 3:**
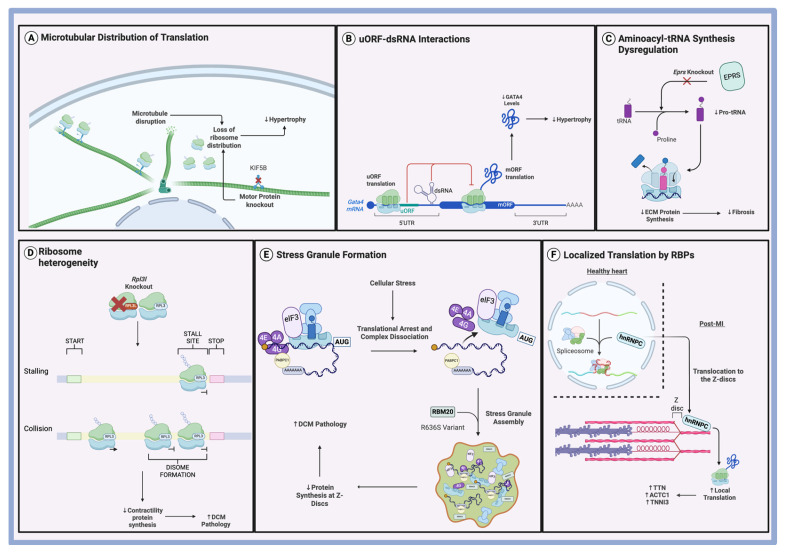
Novel mechanisms of regulation in cardiac pathology. (**A**) Microtubular distribution of translation. Spatial distribution of translating ribosomes in hypertrophying cardiomyocytes requires proper microtubule assembly and functioning motor proteins. (**B**) uORF-dsRNA interactions. uORF translation is enhanced by resident dsRNA on *Gata4* mRNA, inhibiting mORF translation and reducing CM hypertrophy. (**C**) Aminoacyl-tRNA synthesis dysregulation. *Eprs* knockout leads to an inability to synthesize proline-tRNAs, reducing the translation of proline-rich ECM proteins, hence inhibiting fibrosis formation. (**D**) Ribosome heterogeneity. RPL3L ribosomes are necessary for avoiding ribosome stalling and collisions, with *Rpl3l* knockout resulting in decreased synthesis of cardiac contractility-proteins and subsequent DCM pathology. (**E**) Stress granule formation. The pathogenic variant of RBM20 increases the formation of stress granules, which sequester mRNA and translational machinery, resulting in decreased protein synthesis at Z-discs and contributing to DCM pathology. (**F**) Localized translation by RBPs. hnRNPC translocates to the Z-discs during post-MI recovery, where it enhances the local translation of structural proteins.

The protective effect of microtubule disruption suggests that microtubules are crucial for hypertrophic growth by facilitating the necessary distribution of mRNA and ribosomes within cardiomyocytes. Using a methionine analog (HPG) with click chemistry, the researchers showed that while control cells exhibited a decline in newly synthesized protein activity as the distance from the nucleus increased, PE-treated cells displayed uniform protein synthesis across the cell. This shift in translational activity was dependent on intact microtubules, as microtubule disruption led to more centrally-localized translation and a loss of peripheral mRNA and ribosome distribution. In addition, the motor protein Kinesin-1, encoded by *Kif5b*, was found to be specifically required for the positioning of translational machinery in response to PE-induced hypertrophy (Figure 3A). Knockdown of *Kif5b* resulted in mislocalized mRNA and nuclear accumulation of ribosomes, effectively blocking the hypertrophic response to phenylephrine [28]. These findings suggest that microtubules, along with their associated motor proteins, are integral to the spatial regulation of translation necessary for adrenergic-induced hypertrophy.

Taken together, recent work suggests that while pressure overload may rely on shifts in translational efficiency, adrenergic-induced hypertrophy seems to involve the upregulation of ribosomal components and the organization of translational machinery via the microtubule network. Future work that explores the role of the microtubule network in a pressure-overload system would make the distinction between the two methods of hypertrophy induction clearer.

### 3.5. The Role of uORFs in Hypertrophy

Upstream open reading frames (uORFs) are a class of small ORFs, with an initiation codon located within the 5′-leader sequence of an mRNA and a termination codon either upstream or overlapping with the main ORF [15]. These sequences are estimated to be present in 49–58% of human transcripts and have been found in important classes of genes, such as transcription factors and genes involved in growth and differentiation [48]. Upstream open reading frames (uORFs) generally reduce the translation efficiency of downstream genes, limiting the production of proteins essential for cellular growth and homeostasis.

Recent research has highlighted the role of upstream open reading frames (uORFs) in the regulation of gene expression in cardiomyocytes, specifically during hypertrophy. Doroudgar et al. showed that in the heart, under hemodynamic stress, transcripts containing uORFs saw a downregulation of translational activity [6]. One notable example of a transcript translationally downregulated by its uORF in response to hypertrophy is *Flcn* (folliculin). FLCN is a tumor suppressor and was found by Bencun et al. to be an inhibitor of the mTOR pathway, which promotes protein synthesis during hypertrophic growth. Knockout of the gene induced strong mTORC1 activation with hypertrophic growth. Interestingly, mTORC1 activation led to non-canonical axis upregulation, involving TFE3 and TFEB. These transcription factors are known to regulate lysosomal biogenesis and autophagy, and this manifested in the experiment with an increase in lysosomal number and size in the cardiomyocytes [17]. As cardiac remodeling demands significant protein turnover, the upregulation of lysosomal biogenesis aligns with the need for a compensatory response to increased protein synthesis. By promoting the breakdown and recycling of proteins, FLCN aids in maintaining cellular homeostasis during the hypertrophic response. This is an important demonstration of how translational regulation by a uORF can have significant downstream effects on cardiac remodeling through dynamic transcriptional modulation in response to stress.

An added layer of complexity to the function of uORFs is their ability to interact with secondary mRNA structures. These structures act as cis-acting regulatory elements that can influence translation, either by serving as internal ribosome entry sites (IRES) or by forming ribosomal blockades that alter scanning dynamics [49]. In a recent study, Hedaya et al. identified a double-stranded RNA (dsRNA) element within the 5′ UTR *of Gata4* mRNA that cooperates with the uORF initiation codon to enhance uORF translation, which in turn suppresses translation of the main open reading frame (mORF) (Figure 3B) [50]. GATA-binding protein 4 (GATA4) encodes the zinc finger transcription factor, which is an important regulator of cardiomyocyte development and hypertrophy [51,52]. Using antisense oligonucleotides (ASOs) to add a more stable dsRNA structure to the mRNA enhanced uORF translation and decreased GATA4 protein levels, resulting in decreased CM hypertrophy. Conversely, disrupting the dsRNA with ASOs inhibited uORF translation, resulting in increased GATA4 expression and enhanced hypertrophy [50]. These findings illustrate that secondary mRNA structure can modulate uORF activity, thereby indirectly controlling mORF translation and influencing downstream cellular phenotypes such as hypertrophy.

Although uORFs have been shown to play a role in translational inhibition, the precise mechanisms through which they act remain poorly understood. In the Ribo-seq analysis of dilated cardiomyopathy (DCM) hearts, van Heesch et al. found no consistent anticorrelation between the translation rates of uORFs and their corresponding coding sequences (ORFs). For some genes, like *ZMPSTE24* and *EIF4G2*, there was an inverse relationship between uORF and ORF translation, but for many others, this was not the case. The relevance of *ZMPSTE24* is noteworthy because it encodes prelamin A (LMNA), a protein that plays a critical role in maintaining nuclear structure and has been implicated in pathological cardiac dilation [9]. Yan et al. reported similar results in their study on neonatal rat ventricular cardiomyocytes treated with phenylephrine to induce hypertrophy. When comparing the translational efficiencies of ORFs and their corresponding uORFs, they found no discernible pattern or correlation, even in pathways central to hypertrophy, such as PI3K/AKT and MAPK [47]. This suggests that the mechanisms by which uORFs influence translation may be more complex and context-dependent than initially thought.

One potential explanation for the lack of correlation between uORF and ORF translation is that uORF-encoded peptides may function as molecular “roadblocks”, stalling ribosomal movement without necessarily affecting the translation of the downstream ORF in a proportional manner. This model posits that the presence of a uORF may slow or regulate ribosome progression without directly inhibiting translation in all cases [53]. Furthermore, the lack of sequence conservation across uORFs suggests that the regulation they impart may be more dependent on their secondary structure rather than the specific sequence of nucleotides they contain [9]. Further research is needed to fully understand the mechanisms by which uORFs exert their regulatory effects and how they contribute to the translational landscape during cardiac stress, particularly hypertrophy.

## 4. Translational Regulation of Cardiac Fibrosis

Cardiac fibrosis is a pathological outcome of the tissue repair process orchestrated by myofibroblasts. Common to many forms of heart disease, fibrosis is marked by the excessive accumulation of extracellular matrix (ECM) and the activation of fibroblasts into myofibroblasts, a transition primarily driven by transforming growth factor-beta (TGF-β) [54]. The resulting scar tissue reduces cardiac compliance and impairs overall heart function [55]. While the transcriptional landscape of fibrosis has been thoroughly studied, the post-transcriptional and translational regulation of this process remains relatively unexplored [56].

### 4.1. RNA-Binding Proteins in Cardiac Fibrosis

Recent findings have demonstrated translational regulation to be integral to the heart’s fibrotic response. Chothani et al. found that approximately one-third of the genes exhibiting expression changes during fibroblast activation were regulated at the translational level. Notably, RNA-binding proteins (RBPs) such as QKI and MBNL2, which are known for their roles in alternative splicing, were found to regulate the translational efficiency of hundreds of transcripts during fibrosis [7]. These findings demonstrate a pivotal role for these RBPs in controlling ECM deposition, a process believed to be translationally regulated.

Interestingly, Chothani et al. also found a 21% overlap in the targets QKI and PUM2. This finding suggests that multiple RBPs can coordinate to regulate specific mRNAs at the translational level before they are expressed as proteins. In the context of fibrosis, RBPs were found to exert influence predominantly through translational regulation, with minimal changes at the transcript level. Moreover, RBPs were capable of both upregulating and downregulating translation in response to fibrotic signals. Using ribosome profiling of human DCM heart samples, the study authors identified 14 RBPs that act as translational regulators, targeting 926 genes associated with fibrosis. Knockdown experiments for two of these RBPs, PUM2 and QKI, showed a reduction in the fibrotic response to TGF-β stimulation, further underscoring the importance of translational regulation in fibrosis [7].

### 4.2. tRNA Synthesis in Cardiac Fibrosis

Aminoacyl-transfer RNAs (aa-tRNAs) are essential components of the eukaryotic translational machinery. They are formed through the ligation of amino acids to their corresponding tRNAs, reactions catalyzed by aminoacyl-tRNA synthetases [57]. One of these enzymes is glutamyl-prolyl-tRNA synthetase (EPRS), which catalyzes the fusion of glutamic acid and proline to their corresponding tRNAs (Figure 3C) [58]. The relevance of EPRS to fibrosis lies in the fact that collagen and other ECM-related transcripts are dependent on the presence of proline tRNAs for translation [59].

Genetic knockout or pharmacologic inhibition of EPRS has been shown to reduce pathological fibrosis in vivo [60]. Notably, this selective downregulation of translation did not impair global mRNA translation, suggesting a targeted effect on proline-rich transcripts [60]. The effect seems to be specific to the synthesis of proline tRNAs, as inhibition of other tRNA synthetases, such as leucyl-tRNA synthetase, could not produce the same anti-fibrotic effects [60].

Further analysis of transcripts with reduced translation following EPRS loss led to the identification of SULF1 as a novel marker of cardiac fibroblast activation [60]. These findings underscore the idea that enzymes involved in generating the building blocks for protein synthesis can also exert selective regulatory control over translational programs relevant to pathological processes such as fibrosis.

## 5. Translational Regulation of Dilated Cardiomyopathy

Dilated cardiomyopathy (DCM) is characterized by left or biventricular dilation and systolic dysfunction [61]. Its pathophysiology is multifactorial and complex, encompassing a range of contributing factors, including genetic mutations, viral myocarditis, autoimmune responses, and toxic insults such as alcohol or chemotherapeutic agents. Mutations in genes encoding cytoskeletal, sarcomeric, desmosomal, nuclear envelope, and RNA-binding proteins have all been implicated in the development of DCM [62]. Despite the identification of many pathogenic gene mutations, the precise mechanisms linking genotype to phenotype are not well understood. In recent years, growing interest in post-transcriptional regulation has begun to uncover novel insights into the pathogenesis of DCM, offering a deeper understanding of how RNA-based mechanisms may contribute to disease progression.

### 5.1. Protein Truncating Variants in DCM

Similar findings on translational regulation in fibrosis were observed in the ribosome profiling of human hearts with dilated cardiomyopathy (DCM). By comparing the mRNA expression and translation profiles of left ventricular tissue from patients with end-stage DCM and non-DCM controls, in the study by van Heesch et al., the authors found that certain cellular processes, especially ECM production, were preferentially regulated at the translational level [9]. This suggests that, during the progression of DCM, key pathways involved in fibrosis and tissue remodeling are regulated at the translational level.

One important finding from van Heesch et al.’s study was the role of protein-truncating variants (PTVs) in contributing to DCM. These variants often result in inefficient translational termination, as ribosome occupancy analyses before and after the PTVs revealed that only 17.1% of PTVs showed reduced translation. This indicates that translation can continue beyond the truncated regions or initiate downstream of the truncation, contributing to DCM pathology. Titin truncating variants (TTNtv), the most common genetic cause of DCM, were found to exhibit normal premature translation termination in only 4 out of 13 carriers [9]. These results suggest that improper translational termination, particularly of TTNtv, contributes to the development of DCM, emphasizing the pathological consequences of aberrant translational control in cardiomyopathy.

### 5.2. Ribosome Heterogeneity in DCM

The composition of ribosomes can vary across different cell types [63,64,65]. Different ribosomal subtypes are capable of preferentially translating specific subsets of mRNA [66]. Although the concept of tissue-specific ribosomes has been proposed, it remains a topic of active debate [67,68]. In cardiomyocytes, the heterogeneity of ribosomes is especially relevant to heart development. The ubiquitous core ribosomal protein L3 (RPL3), which guards the coding center of the ribosome, is gradually replaced by its paralog RPL3-like (RPL3L) during development [69]. Eventually, RPL3L becomes the dominant form in adult human left ventricular cardiomyocytes [70]. RPL3L is a component of the 60S ribosomal subunit and has been found to be expressed specifically in the heart and skeletal muscle [71]. While similar in structure to its canonical version, RPL3L differs from RPL3 by one-fourth of its amino acid positions [72].

Clinically, mutations in RPL3L have been found in patients with neonatal DCM [73]. Using a *Rpl3l* knockout model, Shiraishi et al. found that RPL3L ribosomes regulate global translational elongation dynamics but have a specifically strong effect on genes related to cardiac muscle contraction and DCM pathology [74]. Loss of RPL3L resulted in specific ribosomal stalling at Ala/Pro codons, delaying translational elongation and resulting in an increased rate of ribosomal collisions (Figure 3D) [74]. Ribosomal collisions are detected by the cell through a ribosome-associated quality control pathway and result in degradation of the translating protein [75]. Through these two mechanisms, ribosomal stalling and ribosomal collisions, cardiomyocytes experienced a reduction in cardiac contractility proteins, which manifested pathologically as reduced LVEF [74].

However, the role of RPL3L remains controversial, as Milenkovic et al. did not observe differences in translational efficiency with *Rpl3l* knockout, instead reporting enhanced ribosome–mitochondria interactions [76]. Grimes et al. found neither changes in translational output nor changes in ribosomal localization [77]. Consolidating these different viewpoints, Shiraishi et al. proposed that differences in mouse strains and developmental stages may account for these discrepancies [74]. Altogether, while these findings highlight a potential role for tissue-specific ribosomes in cardiac physiology and disease, further studies are needed to clarify their functional relevance and mechanistic impact.

### 5.3. Stress Granules in DCM

Stress granules (SGs) are membraneless cytoplasmic organelles composed of stalled pre-initiation complexes, mRNAs, and translation initiation factors [78]. They are formed in response to various cellular stress pathways that halt translation initiation, such as eIF2α phosphorylation, inactivation of the eIF4F complex, or silencing of eRF1 [79]. The assembly and disassembly of SGs is mediated by RNA-binding proteins (RBPs), enabling cells to temporarily arrest translation and rapidly recover once homeostasis is restored [79]. One such RBP is RBM20, a splicing regulator essential for cardiovascular development, which has also been implicated in stress granule dynamics [80]. Utilizing a pig model of DCM with a genomically edited pathogenic R636S allele of RBM20, Schneider et al. demonstrated that the mutation causes the formation of stress granule-like condensates [81]. These granules contribute to DCM pathogenesis by sequestering cytoskeletal mRNAs away from their proper localization at Z-discs, thereby disrupting translation at critical structural sites (Figure 3E) [81]. Historically, RBM20-linked DCM has been attributed to alterations in alternative splicing [82]. However, these new findings reveal an additional mechanism involving post-transcriptional mislocalization of mRNAs. This layer of regulation underscores the complex interplay between mRNA trafficking, localization, and translation in the pathogenesis of DCM.

## 6. Translational Regulation of Ischemic Heart Disease

Myocardial infarction (MI) often progresses to heart failure, a process exacerbated by the limited regenerative capacity of the adult myocardium. The basal rate of cardiomyocyte renewal is extremely low and insufficient to compensate for the extensive cell loss that occurs during ischemic injury [83]. A deeper understanding of the cellular damage sustained by cardiomyocytes during MI can help pinpoint the pathophysiological processes that must be targeted or reversed by therapeutic interventions. At the same time, developing strategies to enhance the regenerative potential of cardiomyocytes is essential for improving outcomes post-MI and slowing the progression to heart failure.

### 6.1. RNA-Binding Proteins in Ischemic Heart Disease

Heterogeneous nuclear ribonucleoprotein C (hnRNPC) is an RNA-binding protein that normally resides in the nucleus and regulates splicing [84]. Under stressful conditions, the protein is capable of translocating to different cellular compartments [85]. In a recent study, Martino et al. found that following myocardial infarction, hnRNPC translocates from the nucleus to the sarcomeres, where it interacts with Z-disc components (Figure 3F) [86]. Notably, in the diseased heart, hnRNPC exhibited reduced association with the spliceosome, suggesting a functional shift away from splicing [86]. Instead, it was found to promote local translation of key structural proteins such as TTN, ACTC1, and TNNI3 at the Z-discs [86]. This study highlights the functional plasticity of RNA-binding proteins (RBPs), revealing that they can shift roles from splicing to translational regulation in response to pathological stress. These findings raise the possibility that many RBPs not currently recognized as translational regulators may acquire such functions under specific disease conditions, warranting further investigation across various pathological contexts.

### 6.2. Mitochondrial Protein Translation in Ischemic Heart Disease

Mitochondrial function plays a crucial role in cardiac regeneration following myocardial infarction. During development, the loss of regenerative capacity in the mammalian heart is accompanied by a metabolic shift linked to mitochondrial maturation [87]. Interestingly, studies have shown that reprogramming metabolism in mature cardiomyocytes can enhance their proliferative potential, suggesting that targeting mitochondrial function could be a promising strategy for improving regeneration [88]. One approach involves modulating mitochondrial protein synthesis to influence mitochondrial performance.

**Figure 4:**
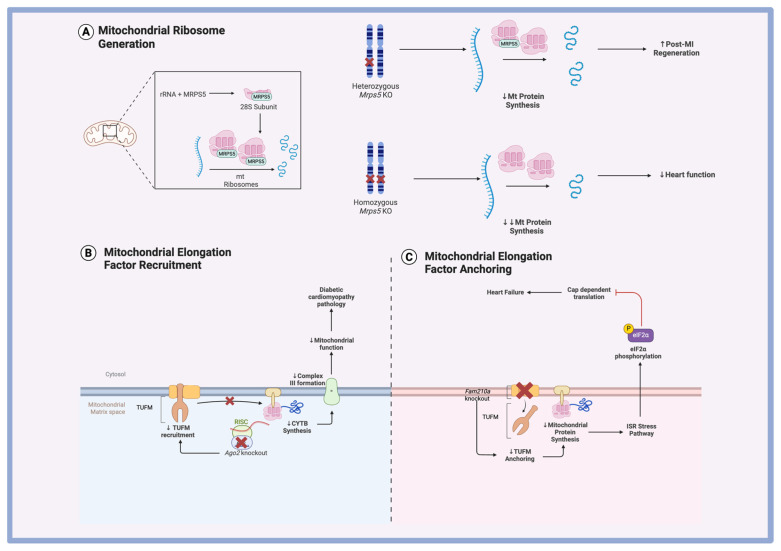
Mitochondrial protein synthesis regulation. (**A**) Mitochondrial ribosome generation. Heterozygous *Mrps5* knockout results in a modest decrease in mitochondrial protein synthesis, resulting in increased regeneration after a myocardial infarction. Homozygous knockout results in more dramatic inhibition of mitochondrial protein synthesis, causing decreased heart function. (**B**) Mitochondrial elongation factor recruitment. *Ago2* knockout prevents TUFM recruitment to mitochondrial mRNA, reducing ETC Complex III protein translation, inhibiting mitochondrial function, and contributing to diabetic cardiomyopathy pathology. (**C**) Mitochondrial elongation factor anchoring. FAM210A is necessary for the anchoring of TUFM to the mitochondrial membrane, and its knockout results in decreased mitochondrial protein synthesis, activation of the ISR stress pathway, and inhibition of cap-dependent translation, manifesting in heart failure.

FAM210A, a mitochondrial protein essential during embryonic development, has emerged as a key player in this process. Wu et al. demonstrated that *Fam210a* knockout results in diminished translation of mitochondria-encoded proteins [89]. Previous findings showed that FAM210A interacts with EF-Tu (or TUFM), a translational elongation factor, to promote mitochondrial protein synthesis (Figure 4C) [60]. Combining these insights, the authors proposed a model in which FAM210A complexes with ATAD3A, acting as a scaffold to increase the local concentration of EF-Tu at the inner mitochondrial membrane and enhance the availability of translational machinery [89]. The disruption of this process results in two main downstream effects. First, the reduction in mitochondrial proteins disrupts mitochondrial function, causing a decreased respiratory rate and increased presence of ROS [89]. Second, the disruption in homeostasis triggers the ISR stress response pathway. This pathway causes the phosphorylation of the eukaryotic translational initiation factor eIF2α, inhibiting its function and leading to a global reduction in cytoplasmic mRNA translation [90]. Notably, overexpression of FAM210A in a mouse myocardial infarction model improved cardiac function, underscoring the therapeutic potential of enhancing mitochondrial translation [89].

Conversely, partial inhibition of mitochondrial translation may also yield regenerative benefits. Gao et al. investigated the role of MRPS5, a component of the small mitochondrial ribosomal subunit, in regulating mitochondrial mRNA translation (Figure 4A) [91]. Homozygous *Mrps5* knockout severely impaired cardiac function due to a complete loss of mitochondrial translation [92]. However, heterozygous knockout produced the opposite effect: it preserved heart function, increased cardiomyocyte proliferation, and enhanced regeneration post-MI [91]. The authors reconciled the two findings by hypothesizing that the reduction in mitochondrial translation must be titrated to ensure a beneficial effect. They proposed that partial inhibition of mitochondrial translation triggers the ISR, leading to ATF4 overexpression, a transcription factor they found essential for promoting cardiac regeneration [91]. These findings support the notion of regulating mitochondrial protein translation as a therapeutic avenue but also emphasize the need to carefully modulate the dosage of translational change to achieve therapeutic benefits without detrimental effects.

## 7. Translational Regulation of Diabetic Cardiomyopathy

Diabetic cardiomyopathy is defined as myocardial dysfunction in diabetic patients that cannot be fully explained by other cardiovascular or systemic conditions [93]. Diabetic cardiomyopathy is characterized by metabolic dysregulation and mitochondrial dysfunction [94]. Mitochondrial abnormalities are believed to play a direct role in promoting oxidative stress and cardiomyocyte death, contributing to the progressive decline in cardiac function observed in diabetic cardiomyopathy [95]. Therefore, understanding how mitochondrial protein translation is altered in diabetes may provide new insights into disease mechanisms.

MicroRNAs (miRNAs), small non-coding RNAs known for their ability to modulate gene expression, have emerged as important regulators in this context. The canonical role of miRNAs involves the inhibition of mRNA translation through binding to the 3′ UTR, but non-canonical functions, such as stimulation of translation, have also been documented [96,97]. Dysregulation of miRNA networks has been implicated in diabetic cardiomyopathy, highlighting their potential role in mitochondrial and metabolic remodeling [98,99]. While several studies have proposed miRNA-mediated translational regulation in diabetic hearts, direct translatome profiling studies have only recently emerged [100,101].

Zhan et al. applied Ribo-seq to investigate translational dynamics in models of diabetic cardiomyopathy and uncovered a perturbed pathway of mitochondrial protein synthesis involving the miRNA-induced silencing complex (miRISC) [102]. Central to this pathway is AGO2 (Argonaute 2), which forms a complex with miRNAs to regulate translation [103]. In late-stage type II diabetic cardiomyopathy, AGO2 expression was significantly reduced, accompanied by a selective decrease in the translation of mitochondria-encoded proteins, particularly components of the electron transport chain complex III such as CYTB (Figure 4B) [102]. The authors proposed that AGO2 facilitates the recruitment of TUFM, a mitochondrial translation elongation factor, to mitochondrial mRNAs (mt-mRNAs), thereby promoting mitochondrial protein synthesis [102]. Notably, the decline in translation was not uniform across all mitochondrial transcripts, as certain proteins were more affected than others, suggesting that specific mt-miRNAs confer translational selectivity via their interaction with AGO2 [102]. Together, these findings highlight a novel layer of translational regulation in diabetic cardiomyopathy, wherein miRNAs and AGO2 modulate mitochondrial protein synthesis in a target-specific manner. This expands our understanding of the molecular underpinnings of diabetic heart disease and suggests potential therapeutic targets in the miRNA–mitochondrial translation axis.

## 8. Translational Regulation of Fibroblast-Cardiomyocyte Reprogramming

Translational regulation has also been shown to play a critical role in cellular reprogramming, particularly in converting fibroblasts to induced cardiomyocytes (iCMs). Xie et al. performed a translational analysis during fibroblast-to-iCM fate conversion and found that several key pathways involved in cell size regulation and cytoskeletal rearrangement were upregulated at the translational level, despite no significant changes in transcription. Conversely, genes associated with ubiquitination and mesenchymal cell proliferation were downregulated translationally [104].

One of the most striking findings in the reprogramming process was the role of the RNA-binding protein YBX1, which has been discussed previously in the context of mTORC1-regulated hypertrophy. Xie et al. identified YBX1 as a blocker of reprogramming, whereby silencing *Ybx1* increased the efficiency of fibroblast conversion into iCM. YBX1 was found to regulate the translation of critical genes, including *SRF* and *Baf60c*, which are involved in cytoskeletal organization and chromatin remodeling. While the transcription levels of these genes did not significantly change following *Ybx1* knockdown, their protein expression was substantially increased, indicating that YBX1 primarily exerts its regulatory effects at the translational level. Further experiments showed that knocking down *Ybx1*, along with either *SRF* or *Baf60c*, attenuated the rescue effect observed during reprogramming, suggesting a reprogramming-block pathway that involves YBX1, SRF, and BAF60C [104]. These findings demonstrate the critical role of translational regulation in cellular reprogramming and highlight new avenues for improving the efficiency of cellular conversion in regenerative medicine.

## 9. Clinical Relevance

Exploring translational regulators as therapeutic targets in the heart is a relatively novel concept, but clinical therapies targeting these mechanisms have yet to be developed. Nevertheless, preclinical models have revealed promising treatment avenues. One of the most straightforward strategies involves the use of mTOR inhibitors. Inhibition of mTOR signaling has been shown in preclinical studies to reduce pathological hypertrophy and improve ventricular function [105]. Clinically, mTOR inhibitors are already used to treat certain types of cancer, but their application in cardiovascular disease has been limited, with little data available on their effectiveness in heart failure [106]. A potential barrier to broader use is their lack of specificity, which may lead to off-target side effects. However, newer, more selective mTOR inhibitors, such as Torin1, have demonstrated reduced toxicity [107]. Preclinical testing of such agents in heart failure models could serve as a valuable first step toward future clinical trials.

As mentioned previously, colchicine has been shown pre-clinically to be protective in hypertrophy by affecting translation in cardiomyocytes through the disassembly of microtubules [28]. Interestingly, in the COLCOT trial, low-dose colchicine was tested in patients following myocardial infarction resulting in a 23% reduction in adverse cardiac events compared to a placebo [108]. Despite these encouraging findings, the potential use of colchicine to specifically target pathological hypertrophy, such as in heart failure, remains largely unexplored.

Lastly, antisense oligonucleotides (ASOs) have shown promise in modulating translation by targeting upstream open reading frames (uORFs). UORFs have shown the capacity to reduce the production of harmful proteins or enhance the translation of beneficial ones [109]. In one study, ASOs improved ventricular filling in a mouse model of heart failure with preserved ejection fraction (HFpEF) by targeting the RNA-binding protein RBM20. These findings were further validated in human iPSC-derived cardiac tissue [110]. Outside of the heart, 15 oligonucleotide-based therapies, several of which are ASOs, have already gained market approval across various countries [111]. Developing targeted ASO therapies for heart disease thus represents a highly promising and emerging area of research.

## 10. Conclusions

**Figure 5:**
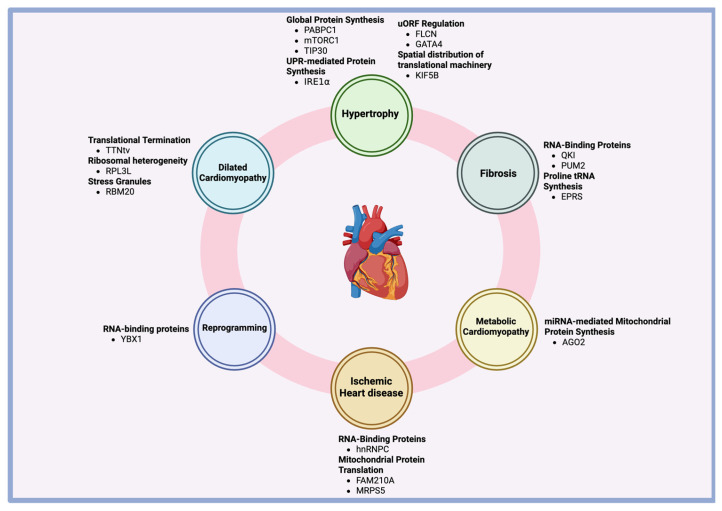
Overview of translational regulation in the heart.

The complex mechanisms governing translation, ranging from ribosomal recruitment, initiation, elongation, and termination, offer cardiomyocytes the ability to precisely modulate protein synthesis in response to mechanical stress, pressure overload, or other pathological stimuli (Table 1). Given the relatively low levels of transcriptional activity in the heart, particularly under stress, the regulation of protein synthesis through translation becomes crucial. Techniques like ribosome profiling have provided insights into the complex landscape of translation in cardiomyocytes, revealing key regulatory proteins like mTORC1, PABPC1, and YBX1, which fine-tune the process of protein production during hypertrophy. Additionally, translational regulation has been found to play roles in the progression of pathologies like fibrosis, dilated cardiomyopathy, metabolic cardiomyopathy, ischemic heart disease, and fibroblast-to-cardiomyocyte reprogramming (Figure 5).

Significant gaps remain in our understanding of translational regulation in the heart. The study of upstream open reading frames (uORFs) and their role in translational control offers a promising research direction. Understanding the mechanisms by which uORFs manipulate translation in cardiac disease could provide valuable therapeutic targets, particularly through their potential to be targeted by antisense oligonucleotides to repress or enhance translation in a sequence-specific manner. 

RNA-binding proteins (RBPs), particularly those traditionally associated with splicing, should be more thoroughly investigated for potential functions in translational regulation. As demonstrated by hnRNPC, RBPs may undergo functional shifts in pathological states, switching from splicing regulation to roles in localized translation. Uncovering similar transitions in other RBPs could significantly expand our understanding of cardiac gene regulation. Additionally, the involvement of microtubules in the spatial regulation of translation in adrenergic versus pressure overload-induced hypertrophy requires further exploration to better understand cytoskeletal contributions to cardiac remodeling. Lastly, further characterization of specialized ribosomes like RPL3L in cardiomyocytes is needed, as current studies offer interesting but opposing insights into their roles. Expanding our knowledge of these regulatory elements will not only provide deeper insights into cardiac physiology but also present novel therapeutic strategies for treating a variety of cardiac diseases.

## Figures and Tables

**Table 1:** Proteins relevant to cardiac translational regulation.

Protein	Function	Related Disease Process	References
PABPC1	Translational initiation and recruitment of the 40S ribosomal subunit	Cardiomyocyte Hypertrophy	[30]
mTORC1	Promoting interactions between translational components during initiation and regulation of elongation through downstream effectors	Cardiomyocyte Hypertrophy	[35,36,37]
IRE1α	Coordination of the assembly of the translational initiation complex (through interactions with eIF3 and eIF4G)	Cardiomyocyte Hypertrophy	[29]
TIP30	Inhibition of elongation factor 1A1 (eEF1A1) to slow translational elongation and protein synthesis	Cardiomyocyte Hypertrophy	[44]
KIF5B	Spatial positioning of translational machinery in response to phenylephrine-induced hypertrophy	Cardiomyocyte Hypertrophy	[28]
FLCN	Inhibition of the protein synthesis pathway (mTOR) during hypertrophic growth	Cardiomyocyte Hypertrophy	[17]
YBX1	RNA- and DNA-binding protein involved in transcription, translation, and mRNA stability	Cardiomyocyte Hypertrophy and Fibroblast–Cardiomyocyte Reprogramming	[41,104]
RBM20	RNA-binding protein and splicing factor with a role in stress granule formation	Dilated Cardiomyopathy	[80,81,82]
RPL3L	Paralog of RPL3. Component of the 60S ribosomal subunit. Regulates ribosomal collisions and interactions between ribosomes and mitochondria	Dilated Cardiomyopathy	[74,76,77]
MRPS5	Regulation of mitochondrial mRNA translation; component of the small subunit of the mitochondrial ribosome	Myocardial Infarction	[91,92]
FAM210A	Anchoring of TUFM for upregulation of mitochondrial protein expression	Myocardial Infarction	[60,89]
hnRNPC	Regulation of splicing. In the diseased heart: Regulation of localized translation at Z-discs	Myocardial Infarction	[86]
AGO2	Recruitment of translation factor (TUFM) to promote mitochondrial protein translation	Diabetic Cardiomyopathy	[102]
EPRS	Synthesis of aminoacyl tRNAs and regulation of pro-fibrotic protein synthesis	Fibrosis	[60]

## Data Availability

Not applicable.
